# The role of CT imaging for management of COVID-19 in epidemic area: early experience from a University Hospital

**DOI:** 10.1186/s13244-020-00957-5

**Published:** 2021-01-29

**Authors:** Vikram rao Bollineni, Koenraad Hans Nieboer, Seema Döring, Nico Buls, Johan de Mey

**Affiliations:** Department of Radiology, Universitair Ziekenhuis Brussel (UZ Brussel), Vrije Universiteit Brussel (VUB), Laarbeeklaan 101, 1090 Brussels, Belgium

**Keywords:** COVID-19, RT-PCR, Chest CT

## Abstract

**Background:**

To evaluate the clinical value of the chest CT scan compared to the reference standard real-time polymerase chain reaction (RT-PCR) in COVID-19 patients.

**Methods:**

From March 29th to April 15th of 2020, a total of 240 patients with respiratory distress underwent both a low-dose chest CT scan and RT-PCR tests. The performance of chest CT in diagnosing COVID-19 was assessed with reference to the RT-PCR result. Two board-certified radiologists (mean 24 years of experience chest CT), blinded for the RT-PCR result, reviewed all scans and decided positive or negative chest CT findings by consensus.

**Results:**

Out of 240 patients, 60% (144/240) had positive RT-PCR results and 89% (213/240) had a positive chest CT scans. The sensitivity, specificity, positive predictive value (PPV) and negative predictive value (NPV) of chest CT in suggesting COVID-19 were 100% (95% CI: 97–100%, 144/240), 28% (95% CI: 19–38%, 27/240), 68% (95% CI: 65–70%) and 100%, respectively. The diagnostic accuracy of the chest CT suggesting COVID-19 was 71% (95% CI: 65–77%). Thirty-three patients with positive chest CT scan and negative RT-PCR test at baseline underwent repeat RT-PCR assay. In this subgroup, 21.2% (7/33) cases became RT-PCR positive.

**Conclusion:**

Chest CT imaging has high sensitivity and high NPV for diagnosing COVID-19 and can be considered as an alternative primary screening tool for COVID-19 in epidemic areas. In addition, a negative RT-PCR test, but positive CT findings can still be suggestive of COVID-19 infection.

## Key points

- A negative RT-PCR test, but positive CT findings are still highly suggestive of COVID-19 infection.
- Chest CT imaging has high sensitivity and high NPV for diagnosing COVID-19.
- The low to moderate specificity of the chest CT scan can be partly explained by initial false-negative RT-PCR cases.

## Introduction

A novel coronavirus, named severe acute respiratory syndrome coronavirus-2 (SARS-CoV-2), causes an acute respiratory infectious disease, which was recently found in humans, commonly known as COVID-19 [1]. A novel coronavirus, named severe acute respiratory syndrome coronavirus-2 (SARS-CoV-2), causes an acute respiratory infectious disease, which was recently found in humans, commonly known as COVID-19 [1]. The World Health Organization (WHO) declared COVID-19 to be a pandemic on March 12th, 2020 [2]. As of July 4th, total cases of 11,053,488 infected patients globally, with 526,260 deaths, had been reported in 210 countries [3]. The common symptoms of patients were fever, dry cough, fatigue and sore throat. The average incubation period of the disease was found to be 5–6 days [1]. The current gold standard reference for the diagnosis of COVID-19 infection is a real-time reverse transcription polymerase chain reaction (RT-PCR) method applied to upper respiratory tract secretions [4]. However, the sensitivity of RT-PCR from swab samples was reported to be about 30% to 70% at the initial presentation [5–7]. Hence, many patients with COVID-19 may not be identified at the initial presentation and pose a significant risk for infecting larger population given the highly contagious nature of the coronavirus. In addition, RT-PCR method does not allow assessing the disease severity. Alternatively, a chest CT scan is an imaging tool for diagnosing pneumonia and relatively easy to perform and provide rapid screening and diagnosis. Recent studies have shown that the CT imaging can demonstrate typical characteristic radiological findings such as multiple ground-glass opacities, patchy pulmonary consolidations and crazy-paving pattern, typically involving peripheral, sub-pleural and basal areas of the lung in COVID-19 patients [5, 7, 8]. To this end, a chest CT scan may aid a faster diagnosis of COVID-19 and assess the severity of the disease. In this study, we evaluated the value of the chest CT scan compared to the reference standard real-time polymerase chain reaction in COVID-19 patients at initial presentation.

## Materials and methods

The institutional review board of our hospital approved this retrospective study, and written informed consent was waived. The electronic records were reviewed and analyzed between March 29th, 2020 and April 15th, 2020. A total of 240 patients (145 male and 95 female with mean age 63, range 15–104) who presented themselves at the emergency department with symptoms of dyspnoea, cough and fever and underwent both chest CT imaging and laboratory RT-PCR assay (pharyngeal swab within an interval of 4 days) were included in this study. Patients with positive chest CT scan and negative RT-PCR test at baseline underwent repeat RT-PCR assay. The conversion of RT-PCR test result from negative to positive or vice versa was analyzed in correlation with the initial chest CT scan. Currently, the RT-PCR test is a reference standard for COVID-19 infection, and the performance of chest CT in diagnosing COVID-19 was assessed simultaneously.

### CT scanning

All patients were referred for medical imaging and scanned on an Apex Revolution CT (GE Healthcare, Milwaukee, USA). The low-dose non-contrast chest CT scan imaging protocol consists of a spiral acquisition with pitch 1, rotation time 0.35 s and auto kVp and mA selection (average dose length product of 149 mGy cm). Images with 1.25 mm slice thickness were reconstructed with deep learning image reconstruction (DLIR) set at medium level and stored in the PACS system.

### CT image analysis

Two board-certified radiologists with experience in chest CT reading (J.D.M and K.N., mean 24 years of experience) evaluated all the CT images on a dedicated radiological workstation (IMPAX, Agfa Healthcare, Belgium) and decided on positive or negative CT findings by consensus. Both radiologists were blinded to the RT-PCR results. The clinical symptoms and epidemiological history of the patients were available for both readers.

### Statistical analysis

The statistical analysis was performed using SPSS software version 26.0 (SPSS Inc. Chicago, IL). Categorical variables were displayed as counts and percentages, whereas continuous variables were reported as mean ± standard deviation (SD). RT-PCR results are considered as a gold standard, the sensitivity, specificity, positive predictive value, negative predictive value and diagnostic accuracy of the chest CT scan were calculated along with 95% confidence interval.

## Results

### General description

A total of 240 patients (95 women and 145 men) were included in this study with a mean age of 63 years (range: 15–104), Table 1. Out of 240 patients, 60% (144/240) had positive RT-PCR results. Of 96 patients with negative RT-PCR results, 72% (69/96) had positive chest CT scans. A total of 89% (213/240) had evidence of abnormal CT findings compatible with viral pneumonia, regarded as positive CT for COVID-19 infection. The median time interval between the chest CT scan and baseline RT-PCR test was 1 day (range 0–1 day).

**Table 1:** Clinical data

Patient demographics	Number of patients (n = 240)	%
Mean age	63	
Years (range)	15–104	
Total patients	240	100
Male	145	60
Female	95	40
PCR positive	144	60
PCR negative	96	40
CT positive	213	89
CT negative	27	11

### CT diagnostic performance

The overall sensitivity, specificity, positive predictive value (PPV) and negative predictive value (NPV) of the chest CT in suggesting COVID-19 were 100% (95% CI: 97–100%, 144/240); 28% (95% CI: 19–38%, 27/240), 68% (95% CI: 65–70%) and 100%, respectively. The diagnostic accuracy of the chest CT suggesting COVID-19 was 71% (95% CI: 65–77%), Table 2.

**Table 2:** Diagnostic performance of chest CT scan for COVID-19 infection with RT-PCR as the reference standard

	Results (n)				Test performance % with 95% CI				
All patients (n = 240)	TP	TN	FP	FN	Sensitivity	Specificity	PPV	NPV	Accuracy
	144	27	69	0	100 (97–100)	28 (19–38)	68 (65–70)	100	71 (65–77)

*TP* true positive, *TN* true negative, *FP* false positive, *FN* false negative, *PPV* positive predictive value, *NPV* negative predictive value, *CI* confidence interval

**Fig. 1:**
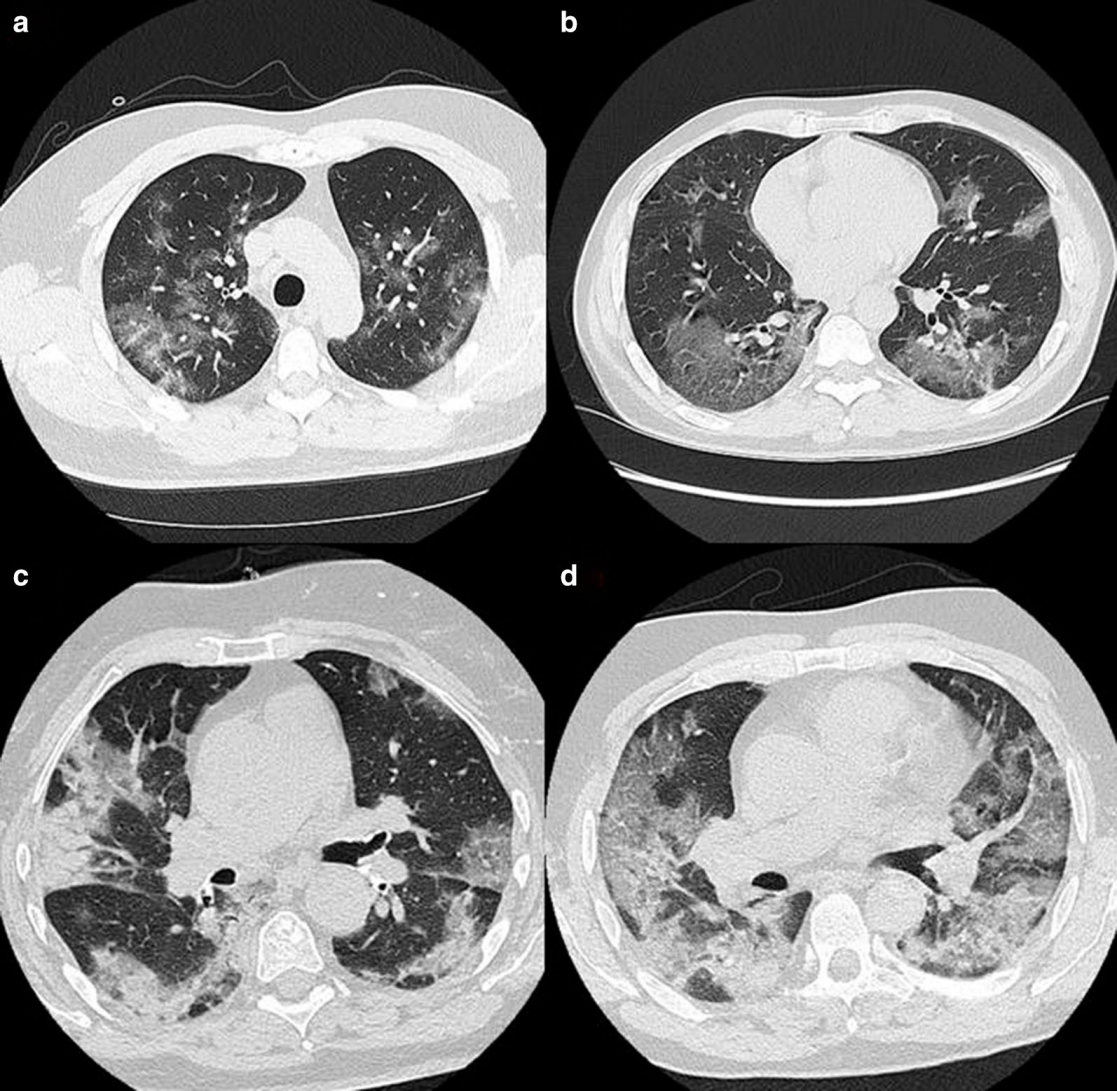
Axial thin-section CT scans in four COVID-19 patients with negative RT-PCR at baseline. **a** 15-year-old male (6 days after symptom onset to CT scan) shows multiple focal ground-glass opacities associated with linear inter and intralobular septal thickening in upper lobes. **b** 54 years old male (10 days after symptom onset to CT scan) shows bilateral and predominantly peripheral consolidation pattern in both lower lobes. **c** 73 years old female (7 days after symptom onset to CT scan) shows extensive bilateral, peripheral, sub-pleural mixed ground-glass opacities in both lower and upper lobes. **d** 56-year-old male (10 days after symptom onset to CT scan) shows predominantly diffuse consolidation in both lungs

A total of 33 patients with negative RT-PCR at baseline underwent repeat RT-PCR assay. In this subgroup, 21% (7/33) patients turned out to be COVID-19 positive. The mean time interval between baseline negative RT-PCR and positive RT-PCR assay was 3 ± 2 days with a range of 2–4 days (n = 7). All seven patients showed typical radiological imaging features compatible with COVID-19 at the baseline chest CT scan. As an example, the description of four patients with negative RT-PCR at baseline and positive chest CT scan results is shown in Fig. 1.

## Discussion

Currently, the reference standard test for establishing the COVID-19 infection is using the RT-PCR assay. However, the recently published literature reports a lack of proper diagnostic sensitivity of the test, which is unfavorable to control the infection in epidemic areas [5, 7, 9, 10]. A noninvasive chest CT imaging is widely available and accessible with high accuracy and speed. Several recent publications showed that the majority of patients with COVID-19 infection had typical characteristic CT imaging features in the disease process [11], such as different grade of ground-glass opacities (predominantly peripheral location), crazy paving pattern and multi-focal organizing pneumonia. Furthermore, a follow-up chest CT scan can accurately reflect the disease evolution and monitor the treatment response [12–16].

In our study, out of 240 patients, 60% (144/240) had positive RT-PCR results. Of 96 patients with negative RT-PCR results, 72% (69/96) still had positive chest CT scans. A total of 89% (213/240) had evidence of abnormal CT findings compatible with viral pneumonia, regarded as positive CT for COVID-19 infection. In our retrospective analysis, the overall sensitivity, specificity, NPV and diagnostic accuracy of the initial CT scan were 100%, 28%, 100% and 71%. The diagnostic performance of the chest CT scan was in accordance with recently published scientific literature using RT-PCR as a reference standard. For example, Ai et al. [5] evaluated the diagnostic value of chest CT scan compared with RT-PCR assay. In their study, they reported a sensitivity of 97%, the specificity of 25% and an accuracy of 72%. Similar results were reported by Caruso et al. [17] who investigated the chest CT features of COVID-19 patients and to compare the diagnostic performance of the chest CT scan with RT-PCR. In their study, they reported high sensitivity (97%), moderate specificity (56%) and accuracy of 72%. Recent meta-analysis studies performed by Hugo et al. [18], Buyun et al. [19], and Hyungin et al. [20] all showed similar results; the baseline chest CT scan offers excellent sensitivity for detecting COVID-19 especially in the severe epidemic setting. However, the specificity is low. Furthermore, care should be taken while interpreting CT images of patients in very early asymptomatic/incubation phase of the infection; it is very likely that CT chest has lower sensitivity in the very early and hyperacute phase of infection.

The low to moderate specificity of the chest CT scan can be partly explained by initial false-negative RT-PCR cases. For instance, Fang et al. [7] compared the sensitivity of the chest CT and RT-PCR test at the initial patient presentation in 51 patients. In their study, 50 patients have evidence of abnormal CT findings compatible with viral pneumonia at baseline. However, the initial RT-PCR test was positive in only 70% (n = 36) patients. The repeat RT-PCR test showed positive in 15 patients (varied between 2 and 4 repeat RT-PCR tests). Hence, the authors concluded the detection rate of baseline chest CT was highly sensitive (98%) than baseline RT-PCR (71%). Also, in our study, 33 patients with negative RT-PCR at baseline underwent repeat RT-PCR assay. In this subgroup, 21% (7/33) cases turned COVID-19 positive and exhibited typical radiological imaging features compatible with COVID-19 at the baseline chest CT scan (Fig. 1). In the subgroup analysis, the remaining 26 patients (79%) do exhibit ground-glass consolidations (peripheral, subpleural and random distribution). Unfortunately, no further analysis was carried out to identify the etiology of the disease in the subgroup. Indeed, the typical radiological findings of COVID-19 may overlap with those of other viral types of pneumonia. The predictive value of the chest CT scan will therefore depend on the phase of the epidemiological situation and the prevalence of other viruses such as influenza.

Similarly, Ai et al. [5] performed multiple RT-PCR tests and chest CT scans on 1014 suspected COVID-19 cases. 88% (888/1014) of patients had positive chest CT scans, while only 59% (601/1014) of patients were RT-PCR positive. Most importantly, in their study, 93% of all patients whose RT-PCR became positive after a baseline negative test outcome had baseline CT findings compatible with COVID-19. Hence, a patient with a negative RT-PCR test but positive chest CT findings can still be highly suggestive of COVID-19; this certainly has clinical and community risk implications. For example, early detection of COVID-19 patients may allow better control of the virus spread. Secondly, in these false-negative cases, repeat swab tests and patient isolation should be considered. The variable reasons for the low sensitivity of the RT-PCR test may include (1) insufficient viral load in the specimen; (2) laboratory error: (3) improper clinical sampling; (4) performance of the diagnostic detection kits and (5) specimen sampling time (disease development over time). Hence, caution should be exercised while interpreting the results of the RT-PCR assay at baseline.

There are several limitations in the present study (1) inherent selection bias of this retrospective study; (2) limited sample size; (3) this study included only patients with respiratory distress who presented at the emergency department; (4) the chest CT scan reading for COVID-19 detection was done by consensus which does not reflect inter-reader variability; (5) RT-PCR test is not perfect, low positive rate as the reference standard [5] and finally (6) some false-positive cases with typical CT findings but negative RT-PCR test may not exclude COVID-19 infection, and further studies are necessary to confirm the actual COVID status of the patient using serial RT-PCT tests.

## Conclusion

In summary, chest CT imaging has high sensitivity and high NPV for diagnosing COVID-19 and can be considered as an alternative primary screening tool for COVID-19 in severe epidemic areas. The specificity of the chest CT for COVID-19 patients may be underestimated considering relatively low sensitivity of the reference standard RT-PCR assay at baseline, hence a negative RT-PCR test, but positive CT findings are still highly suggestive of COVID-19 infection. In these patients, repeat RT-PCR test and patient isolation should be considered. Our experience during the epidemic outbreak and the post-epidemic phase confirms that the chest CT scan is mainly useful during an epidemic outbreak and in symptomatic patients. From our study, we can conclude that the value of the chest CT scan as a screening tool lies principally in its negative predictive value.

## Data Availability

The datasets used and/or analyzed during the current study are available from the corresponding author on reasonable request.

## Abbreviations

COVID: Coronavirus disease
NPV: Negative predictive value
PPV: Positive predictive value
RT-PCR: Real-Time polymerase chain reaction
SARS-CoV-2: Severe acute respiratory syndrome coronavirus-2
